# A comparison between parent and grandparent dietary provision, feeding styles and feeding practices when caring for preschool-aged children

**DOI:** 10.1016/j.appet.2021.105777

**Published:** 2021-10-27

**Authors:** C. Marr, P. Breeze, S.J Caton

**Affiliations:** 1 Public Health, School of Health and Related Research (ScHARR), University of Sheffield, Sheffield S1 4DA, UK; 2 Health Economics and Decision Science, School of Health and Related Research (ScHARR), University of Sheffield, Sheffield S1 4DA, UK

**Keywords:** Parents, Grandparents, Dietary Provision, Feeding Styles, Feeding Practices, Preschoolers

## Abstract

Grandparents are frequently called upon to provide childcare to young children. Consequently, grandparents may influence the development of children’s eating habits and preferences and may require support with their approach to feeding young children. However, research into grandparental feeding behaviours is scarce. Understanding how grandparental feeding behaviours compare to parental feeding behaviours will further help to establish whether grandparents require specific interventions unique to the grandparental role or if current strategies that target parental feeding behaviour are also appropriate for grandparents. The aim of the present study was to explore the similarities and differences between parent and grandparent dietary provision, feeding practices and feeding styles to preschool-aged children. 72 parents and 44 unrelated grandparents of children aged 2-4 years old took part in an online study and completed an online 24-hour dietary recall using myfood24^®^ to assess dietary provision. Parents and grandparents were providing meals high in saturated fat and sodium and providing below recommended amounts of fruit and vegetables. Overall, feeding practices were similar between parents and grandparents. Although, grandparents scored lower on using food as a reward (p <0.05) and creating a healthy food environment (p < 0.05) compared to parents. Whereas, parents scored higher for promoting balance and variety (p< 0.05). A range of feeding styles were found within each caregiver type, with no significant associations found between caregiver type and feeding style (p>0.05). Strategies to promote healthy eating in young children should be expanded to also target grandparents who act as informal caregivers to preschool-aged children. However, since very few differences in feeding behaviour were reported the content of such strategies may not need to be adapted specifically for grandparents.

## 1 Introduction

The most recent UK survey “Childcare and Early Years Survey of Parents” ^1^ showed that 76 % of children aged 0-4 years old had received some form of childcare during their most recent school term-time week. 64% of children were in receipt of formal childcare (e.g., nursery, childminders) and 33% in receipt of informal childcare (e.g., family, friends). On average, children spent around 19 hours per week in formal childcare, and 10 hours per week in informal childcare. Given that a large proportion of preschool children are receiving some type of childcare, this suggests that children consume a significant proportion of their daily food intake outside of the home environment.

Grandparents are relied upon the most as informal carers ^1–6^, particularly for preschool aged children^3^. For instance, in a survey of 50,000 European adults aged 50 or over, 38% of them provided grandparental care, with 28% providing this weekly or more frequently^7^. Like parents, grandparent’s assume the role of gatekeeper for children’s eating; shaping children’s eating environment and making dietary decisions that influence children’s consumption ^8,9^. Grandparents may be particularly influential during the preschool years as this is a time when children’s eating preferences and habits are formed that can track into adulthood ^10–13^. It is therefore important that we understand the strategies that grandparents are using when feeding their grandchildren and the positive and negative influence they may have on preschoolers’ dietary intake.

Research into caregiver feeding behaviour suggests that caregivers influence children’s dietary behaviours in a complex manner. A caregiver’s feeding style, defined as the emotional environment that caregivers create when a child is eating can have a positive or negative influence on children’s eating behaviour ^14^. Feeding styles reflect how caregivers encourage eating and by how much ^15^ and caregivers have been categorized as having one of four feeding styles; indulgent (placing low demands on a child’s eating but being highly responsive to a child’s wants and needs), uninvolved (placing low demands on a child’s eating with low responsiveness to a child’s wants and needs), authoritarian (placing high demands on a child’s eating with a low responsiveness to a child’s wants and needs) and authoritative (placing high demands on a child’s eating and being highly responsive to a child’s wants and needs), with an authoritative feeding style considered optimum for promoting the best dietary quality and for fostering autonomy in preschoolers ^16,17^. The feeding practices caregivers use are also important and occur within the context of the feeding style ^18^. Feeding practices are the goal-oriented behaviours that caregivers use to directly influence a child’s intake and include, for example, modelling healthy eating, putting pressure on a child to eat, restricting specific food or drink items or monitoring what and how much a child consumes. Some of these practices lead to the caregivers’ desired behaviour, for instance, modelling fruit and vegetable consumption can facilitate child liking and consumption of fruit and vegetables ^19^ whereas others, such a pressuring a child to eat can lead to undesired behaviours like food refusal ^20^.

Strategies to improve young children’s eating therefore often focus on caregivers feeding styles and feeding practices ^21–23^, however they also target caregiver’s provision of food and drinks. Interventions have been designed to encourage caregiver’s to change what they provide as snacks to preschool aged children ^24^ and wider public health campaigns emphasise changes to dietary provision. For instance campaigns encourage caregivers to provide 5 portions of fruit and vegetables per day ^25^, to swap the high sugar items that they might usually provide for low sugar alternatives^26^, provide age appropriate portion sizes ^27^ and limit the number of packaged snacks they provide per day^28^. These strategies typically target and reach parents; whether these strategies are also relevant and appropriate for grandparents is unknown. Understanding grandparent’s feeding behaviours, in the context of parental feeding behaviours will help to establish whether current strategies that target parental feeding behaviour are also appropriate for grandparents. Conversely, if there are stark differences between parents and grandparents feeding behaviours with preschoolers, grandparents may require a more directed approach based on their unique feeding behaviours in the grandparental role.

In previous studies, parents have criticized grandparents for undermining their efforts to provide a healthy diet to their young children, complaining that grandparents are indulgent with their grandchildren, serve them high energy dense foods, in large quantities ^29–33^ and use food as a reward for behaviour ^34^. However, a review of grandparental feeding practices, dietary provision and feeding styles when caring for their preschool aged grandchildren highlighted several methodological shortcomings of the published literature ^9^. First of all, no study has captured all food that grandparents provide to their preschool aged grandchildren whilst in their care. Instead, studies have focused on particular food types such as measuring snack provision or fruit and vegetable provision in isolation. Grandparents who care for their preschool aged grandchildren seem to regard meal times as important and place an emphasis on their role in providing nutritious and homecooked meals to their grandchildren ^29,35^. This suggests that we also need to capture grandparents’ dietary provision of meals as well as focusing on specific food types. Secondly, a substantial amount of evidence regarding grandparental feeding behaviours stems from parental reports^9^; considering that grandparents frequently provide care when parents are absent, it is necessary to capture grandparental feeding directly from grandparents. Finally, although there is a consensus across the literature that grandparents are indulgent with preschoolers, no study has used a validated tool to measure grandparental feeding styles. Using a validated tool would give a more reliable indication of the feeding styles that grandparents are adopting with their preschool-aged grandchildren and explore how their feeding styles may compare with those of parents.

There is also evidence to suggest that the amount of time grandparents spend caring for their grandchildren may influence their feeding behaviours. For instance a UK study focusing on caregivers of children aged 2-8 years old found that grandparents’ feeding practices aligned more closely with those of parent’s the more time they spent caring for their grandchildren^36^. Grandparents who cared for their grandchildren a greater amount were more likely to use practices that foster positive eating in children such as teaching about nutrition and modelling healthy food intake than those who spent less time with their grandchildren. This suggests that grandparent’s may assume more of a parental role when providing frequent care to their grandchildren rather than adopting the stereotypical indulgent grandparent behaviours. However a study conducted in Australia found that the number of hours of care provided by grandparents to children aged 3-14 years old was positively associated with children’s consumption of not only fruit and vegetables but also sweet foods, salty snacks and sugary drinks ^37^. Further research is needed to explore how grandparent’s feeding practices, feeding styles and dietary provision may differ depending on the amount of time they spend caring for their grandchildren.

The aim of the present study is to explore the similarities and differences between parent and grandparent dietary provision, feeding practices and feeding styles to preschool-aged children. We also assess whether grandparental dietary provision, feeding practices and feeding styles, vary depending upon the amount of time they spend caring for their grandchildren.

## 2 Methods

### 2.1 Participants

Parents and grandparents of children aged 2-4 years old were recruited online from across the United Kingdom. Participants in the two groups were distinct and unrelated. Grandparents were eligible to participate if they reported caring for their grandchild at least one day per week and providing at least one meal (not including snacks) to their grandchild on those days. Grandparents who were sole carers of their grandchildren were excluded from the study as primary caretaking grandparents may have a different feeding relationship with their grandchildren than grandparents who act as informal caregivers^36^. If parents/grandparents cared for more than one child in the age range they were asked to complete the survey for just one child. Informed consent was obtained from all participants and the study was reviewed and approved by the School of Health and Related Research ethics committee at the University of Sheffield (application number: 026363).

### 2.2 Procedure

A cross-sectional observational study design was utilised using online methods. This allowed participants to complete the study in their own home, increasing ecological validity ^38^. Participants were invited to take part in the study via advertisements posted on different online platforms including MumsNet (www.mumsnet.com), GransNet (www.gransnet.com), The University of Sheffield volunteers list and Facebook between May 2019 and February 2020. Online methods of recruitment were considered the optimum approach for the study due to being logistically simpler, lower cost, and often achieving higher recruitment rates than offline methods ^39^. After consenting to participate, participants completed an online survey hosted by Qualtrics (Qualtrics, Provo, UT. Version 2019-2020). The survey included 49 items to assess caregiver feeding practices, 19 items to assess feeding styles and 13 demographic questions. Additionally, grandparents completed five questions to assess the amount of time they usually care for their grandchildren per week and their usual provision of meals and snacks per day. Upon completion of the survey, participants completed an online 24-hour food recall to record their food provision to their child/grandchild over a 24-hour period when they were in their care. Upon completion of the survey and food record, participants were provided with a £10 high street e-voucher for their time.

### 2.3 Materials and Measures

#### 2.3.1 Demographics

Demographic data was collected for both the caregiver and child. This included caregiver gender, ethnicity, household income, self-reported weight and height in addition to child gender, caregiver-reported child weight, height and month and year of birth. Grandparents were asked additional questions on the number of days and hours they care for their 2-4-year-old grandchild per week and their typical provision of meals and snacks on those days.

#### 2.3.2 Dietary Provision

A single online 24-hour recall was used to assess caregiver’s food and drink provision to their 2-4-year-old child/grandchild. It did not assess children’s dietary intake since the focus of this work is dietary provision. For grandparents, this included only the food grandparents provided within the 24-hour window and therefore did not always include all food and snacks provided to the child over the 24-hour period, including that provided by other caregivers. This method was chosen as 24-hour recalls are easy to administer, impose less burden on participants time than weighed food diaries and have been used successfully by caregivers of preschool-aged children^40^. The recall was completed using myfood24® ^41^, a UK online dietary assessment tool which overcomes some of the traditional challenges of 24-hour recalls by assisting with both portion size estimation and memory ^42^. The dietary analysis was completed within myfood24® using a large composition table of UK food and drinks. This data was extracted into SPSS for analyses.

#### 2.3.3 Feeding Practices

Caregiver feeding practices were evaluated using the Comprehensive Feeding Practices Questionnaire (CFPQ) ^43^. This uses 49 items to measure twelve different feeding practices. As well as being previously used to assess the feeding practices of parents of children aged two to four ^24,44^ it has also been successfully used to assess the feeding practices of grandparents to their grandchildren ^36,45^. Despite the CFPQ being designed for parents, the wording was adapted for grandparents asking them to focus on their grandchild rather than child. Prior to conducting any analyses, the internal consistency of the scale was assessed for grandparents and parents using Cronbach’s alpha. For grandparents all alphas were deemed good or acceptable for the sample (child control =0.55, emotion regulation = 0.73, balance and variety = 0.67, food as a reward = 0.82, involvement = 0.65, modelling = 0.62, monitoring = 0.91, pressure = 0.74, restriction for health = 0.68, restriction for weight = 0.72, environment = 0.66, teaching about nutrition = 0.59). For parents, all alphas were deemed good or acceptable except for teaching about nutrition, therefore, results based on this subscale are treated with caution (child control =0.73, emotion regulation = 0.85, balance and variety = 0.65, food as a reward = 0.83, involvement = 0.73, modelling = 0.72, monitoring = 0.93, pressure = 0.75, restriction for health = 0.76, restriction for weight = 0.81, environment = 0.67, teaching about nutrition = 0.09).

#### 2.3.4 Feeding Styles

Caregiver feeding styles were measured using the Caregiver Feeding Styles Questionnaire (CFSQ) ^14^. This is a 19-item validated tool and is the most frequently used tool to assess the feeding styles of caregivers ^46^. It has been widely used to assess caregiver feeding styles of children below the age of five ^47–49^. Scores on two dimensions of responsiveness and demandingness are calculated and a sample median split for each is used to categorise caregivers into one of four feeding styles; uninvolved (low demandingness, low responsiveness), authoritative (high demandingness, high responsiveness), authoritarian (high demandingness, low responsiveness) or indulgent (low demandingness, high responsiveness).

## 3 Data Analysis

A power calculation for this study was not carried out given the novelty of this exploratory study. No data were available to extract the relevant information to derive an exact sample size. Therefore, the sample size is modest, and the results should be interpreted with caution.

### 3.1 Dietary Provision: Analysis of Meals

#### 3.1.1 Nutrient level

myfood24^®^ calculates the amount of nutrients for each food and drink item recorded in the food diary. Data was separated by meal type (breakfast, lunch, evening meal, snacks and drinks) at the point of data entry. Therefore, to calculate the nutrient content of each meal, the nutrient quantities for each food item were aggregated using the sum function to create totals for each nutrition element including energy (kcal), total sugar (g), saturated fat (g) and sodium (mg) for breakfast, lunch and the evening meal. Mann-Whitney U tests were used to compare the nutritional content of each meal provided by parents versus grandparents. Each nutritional element (dependent variables) was analysed separately and caregiver type was input as the independent variable. Meal occasions were also analysed separately, rather than comparing total daily provision due to the variation in the number of meals a parent/grandparent provided to the child over the 24-hour period. Conducting the analysis separately for meal type also accounts for the variation in provision between meal type.

#### 3.1.2 Food level

To assess the difference between parents’ and grandparents’ fruit and vegetable provision, the total grams of fruit and total grams of vegetables were calculated for each meal occasion. As myfood24^®^ does not provide a breakdown of the grams of fruit and vegetables served when composite meals have been input into the software e.g. lasagne, this only included cases where fruit and vegetables were input separately for each meal or where a recipe was provided. Cases where a composite meal was input into the software were treated as missing values and excluded from this analysis. Mann-Whitney U Tests were used to explore any differences between parents and grandparents’ provision of fruit and vegetables at mealtimes, conducting separate analyses for each meal occasion.

### 3.2 Dietary Provision: Analysis of snacks

#### 3.2.1 Nutrient Level

To explore the difference between the nutritional content of parent and grandparent snacks provided to 2-4-year olds the average content of energy (kcal), total sugar (g), saturated fat (g) and sodium (mg) of snacks were calculated for each individual participant. This was to account for the difference in the numbers of snacks provided by participants across the 24-hour period as some participants provided more snacks than others. Mann-Whitney U tests were used to compare the nutritional content of snacks provided by parents and grandparents.

#### 3.2.2 Food Level

To explore differences in the content of snacks at the food level, two separate analyses were conducted. First, three new binary variables were created to indicate whether snacks included 1) fruit, 2) vegetables and 3) discretionary food items (biscuits, cakes, chocolate or savoury snacks). Chi-squared analyses were used to statistically compare any differences between parents and grandparents’ provision of these three food types. Second, the average weight (in grams) of fruit, vegetables and discretionary food items in snacks were calculated per participant. Mann-Whitney U tests were used separately for each food item to compare the weight in grams of fruit, vegetables and discretionary food items provided by parents and grandparents.

### 3.3 Feeding Practices

Continuous scores were calculated for each of the 12 feeding practices. Mann-Whitney U tests were used to compare the feeding practices of parents with grandparents. Non-parametric tests were used to explore feeding practices as the data were predominantly non-normally distributed.

### 3.4 Feeding Style

Based on responses to the 19 items, scores on two dimensions of responsiveness and demandingness were calculated. A sample median split for each of these dimensions was used to categorise caregivers into one of four feeding styles; uninvolved (low demandingness, low responsiveness), authoritative (high demandingness, high responsiveness), authoritarian (high demandingness, low responsiveness) or indulgent (low demandingness, high responsiveness). To compare the feeding styles of parents and grandparents a Pearson’s chi-squared test was conducted.

### 3.5 Associations with time spent caring for grandchildren

Analysis of variance was used to explore any associations between grandparents feeding styles and the number of hours grandparents spent per week caring for their preschool aged grandchild. Spearman’s Rho tests were used to explore correlations between grandparents’ feeding practices and dietary provision in both meals and snacks (nutrient level and food level) with the number of hours grandparents spent per week caring for their preschool aged grandchild.

All analyses were performed in SPSS version 26. Significance was established at p < 0.05

## 4 Results

### 4.1 Demographic Characteristics

222 parents and 86 grandparents (total 308) consented to participate in the study. 192 participants (62%) dropped out part way through completion; 53 (17%) participants did not complete the survey and 139 (45%) participants completed the survey but not the food record. This resulted in a final sample of 116 (38%) who completed both the online survey and food record in its entirety; 72 parents (32%) and 44 (51%) grandparents.

The mean age of grandparents was 61 ± 6 years and the mean age of parents was 31 ± 5. 100% of parents and 86% of grandparents were female (Table 1). Both parents and grandparents were predominantly white British (85% and 91%) and had a mean BMI in the overweight category (26.0 ± 6.6 and 26.8 ± 5.1 respectively). 40.2% of grandparents and 40.3% of parents had a household income below £30,000. Most children were of a healthy weight (32%) (BMI z-scores were calculated using the WHO anthropometric calculator http://www.who.int/childgrowth/software/en/), female (58%) and aged 3 (44%). Grandparents cared for their 2-4-year-old grandchildren on average 15.7 ± 8.7 hours per week.

### 4.2 Dietary Provision: Meals

Mann-Whitney U tests revealed no significant differences between the nutritional content of breakfast, lunch or the evening meal served by parents compared to those served by grandparents (Table 2).

Descriptive statistics revealed that on average, parents served more fruit at breakfast and the evening meal than grandparents (breakfast; 23g vs 20g, evening meal; 20g vs. 19g) but less fruit than grandparents at lunch (27g vs, 29g). Grandparents served more vegetables at lunch time than parents (25g vs 13g) and less vegetables at breakfast (0g vs. 0.3g) and the evening meal (44g vs. 49g.) Mann Whitney U tests revealed that there was a significant difference in the amounts of vegetables served at lunch time between parents and grandparents (U=1858, p<0.05). Grandparents served significantly more grams of vegetables at lunch than parents (25g vs 13g). No other significant differences were found between the amounts of fruit and vegetables served at mealtimes by parents compared to that served by grandparents (Table 3).

### 4.3 Dietary Provision: Snacks

As some participants provided more snacks than others across the 24-hour period, the average content of kcal, total sugar, saturated fat and sodium of snacks was calculated for each individual participant based upon the number of snacks offered by the caregiver over the 24 hour period.

The energy content of snacks provided by parents was 110kcals, slightly lower than that provided by grandparents (120kcals). Both parents and grandparents served snacks containing, on average, 11g of sugar and similar amounts of saturated fat (2g and 3g respectively). The sodium content of snacks provided by grandparents was slightly higher (106mg) than parents (93mg). When comparing parent and grandparent provision, no significant differences were found between the nutritional content of parents’ and grandparents’ snacks provided to 2-4-year olds (Table 4).

66% of parents and 61% of grandparents provided fruit as part of a snack. Only 4% of parents and 11% of grandparents provided vegetables as part of a snack. 64% of parents and 61% of grandparents provided discretionary food items as part of a snack. Chi-squared tests revealed no significant differences between the snack content provided by parents and grandparents (p>0.05). (Table 5)

No significant differences were found between the amount of fruit, vegetables or high energy-dense foods served as a snack was observed between parents and grandparents (Table 6). When parents served fruit to their two to four-year-old children as part of a snack the average amount was 38g, for grandparents it was 33g. The amount of vegetables served was smaller for parents and grandparents, 1.0g and 4.0g respectively. Parents and Grandparents both served an average of 14g of cakes, biscuits, chocolate and savoury snack items. Independent sample t tests found no significant differences between the amounts of fruit, vegetables and discretionary food items served by parents compared to grandparents.

### 4.4 Feeding Style

The largest percentage of grandparents had an indulgent feeding style (41%) followed by authoritative (23%), uninvolved (18%) and authoritarian feeding style (18%). The largest percentage of parents had an authoritarian feeding style (42%) followed by indulgent (28%), uninvolved (16%) and authoritative (14%). The results from the chi-squared test showed there was no significant association between caregiver type and feeding style X^2^ (3, N=116) = 7.309, p = 0.063. (Table 7)

### 4.5 Feeding Practices

For both parents and grandparents, the most highly reported feeding practice was promoting balance and variety, and the least reported was using food to regulate emotions as well as restricting food for weight reasons in parents. When comparing parents and grandparents feeding practices, grandparents were significantly more likely to report creating a healthy eating environment (4.1 vs 3.6, p<0.05) than parents. Grandparents were significantly less likely to report using food as a reward (2.2 vs 2.8, p<0.05) and promoting balance and variety than parents (4.5 vs 4.7, p<0.05). There were no significant differences between grandparents and parents for the other feeding practices. (Table 8)

### 4.6 Associations between grandparental feeding behaviours with the amount of time Grandparent’s spent caring for their grandchildren per week

#### 4.6.1 Dietary Provision

Spearman’s correlations indicated that there were no significant correlations between grandparent’s provision of nutrients or fruit and vegetables at mealtimes and the amount of time grandparents spent caring for their grandchild each week. For snacks, there was a significant positive correlation (p<0.05) between amount of vegetables grandparents served as part of a snack and the amount of time grandparents spent caring for their grandchild each week. No other significant correlations were found between grandparents’ snack provision and time spent caring for their grandchild each week. (Table 9)

#### 4.6.2 Feeding Style

Grandparents who used an authoritarian feeding style cared for their grandchildren the most hours per week (mean 19.1 hours) whereas grandparents who used an uninvolved feeding style and indulgent feeding style cared for their grandchildren the least number of hours per week (mean 12 and 13.6 respectively). ANOVA revealed there were no statistically significant differences in the mean number of hours grandparents spend caring for their grandchildren and the different feeding styles used by grandparents F(3,40) = 1.466, p = 0.238. (Table 10)

#### 4.6.3 Feeding Practices

Spearman’s correlations indicated that there were no significant correlations between grandparent’s feeding practices and the amount of time grandparents spent caring for their grandchild each week.

**Table 11:** Spearman’s rho correlations between grandparent’s feeding practices and time spent caring for grandchild each week

	No. of hours
**CFPQ subscale**	
Child Control	0.253
Emotion Regulation	0.136
Food as a Reward	0.101
Balance and Variety	0.155
Involvement	0.172
Modelling	0.074
Monitoring	-0.096
Pressure	-0.177
Restriction for Health	0.208
Restriction for Weight	0.118
Teaching about Nutrition	-0.142
Environment	-0.113

* Significant p<0.05

## 5 Discussion

This study showed that the dietary provision, feeding styles and feeding practices of grandparents who act as informal caregivers to their preschool aged grandchildren were comparable to those of non-related parents of preschool children confirming that grandparents are also a key target for intervention aimed at improving the dietary intake of young children. Moreover, the overall similarity found between parent and grandparent feeding suggests that the content of strategies and public health messages that target the feeding behaviours of parents may also be appropriate for grandparents.

### 5.1 Dietary Provision

Dietary provision of parents and grandparents were similar for both meals and snacks. Nutrient provision at meal times suggest that over the course of a day it is likely that children would exceed their recommended daily intakes of saturated fat (less than 10% of total energy intake ^50^), and sodium (800mg for children aged 1-3 and 1200g for children aged 4 to 6 ^51^) when cared for by either parents or grandparents. Additionally, for both caregiver types, only the evening meal contained a full recommended portion of vegetables for a preschool aged child (40g) and parents and grandparents often failed to provide a full portion of fruit (40g) with any meal ^52^. Considering that it is recommended that a third of each meal be made up of fruit and vegetables ^53^, both caregiver types are providing meals to preschoolers that fall short of these recommendations. Nevertheless, these results are important because they confirm

that public health campaigns and interventions that encourage parents to limit the amount of salt and saturated fat and increase the amount of fruit and vegetables provided to preschoolers could be altered to also engage grandparents and other family members who act as informal childcare providers.

The overall similarity between parents’ and grandparents’ dietary provision conflicts with the accounts of grandparental dietary provision described by parents in the literature. For instance, parents complain that grandparents provide large portion sizes to their preschool aged grandchildren ^31,34^ yet in the current study similar amounts of energy was provided in meals by grandparents and parents. Additionally, parents report that grandparents serve large amounts of high fat and high sugar foods to their preschool aged grandchildren ^32^ yet, on average the amount of cakes, biscuits, chocolate and savoury snacks served by grandparents as part of a snack was similar to that served by parents.

Differences were seen between parents’ and grandparents’ provision of vegetables at the lunch time meal whereby, grandparents served significantly more vegetables than parents (25g versus 13g), albeit still less than the guideline portion size of 40g. Previously, studies have reported conflicting findings on whether parents and grandparents served fruit and vegetables at meal times ^54,55^. Speirs et al. found that grandparents in the U.S were more likely to serve fruit in an evening meal than parents but identified no differences in whether they served vegetables ^54^. In contrast Yue et al. found that grandparents in China were less likely than parents to provide fruit and vegetables ^55^. When fruit and vegetables were provided by both caregiver types as snacks, the amount was far below the guideline amounts for the age range ^52^. Moreover, public health campaigns in the UK ^56^ currently recommend limiting children’s snacks to under 100 kcal per snack up to a maximum of two snacks per day; the snacks provided by both parents and grandparents exceeded this calorie recommendation. This is unsurprising since caregivers of preschool aged children often provide larger than recommended snack portion sizes of high energy dense foods ^57^.

### 5.2 Feeding Practices

Overall, feeding practices were similar between the unrelated parents and grandparents recruited for this study, except for creating a healthy food environment, using food as a reward, and promoting balance and variety. Grandparents were more likely to report creating a healthy feeding environment and less likely to report promoting balance and variety, and using food as a reward than parents. Despite the parents and grandparents recruited in the current study being unrelated, the differences that did emerge are consistent with previous work. For instance, in a UK study of 50 parents and 50 grandparents, grandparents were also more likely to report creating a healthy feeding environment and less likely to report promoting balance and variety than parents^36^. Grandparents may be more able to create a healthy feeding environment than parents as they often have more time and money to do so and therefore can ensure healthy options are available ^35^.

Some discrepancies were found between the current study and that conducted by Farrow ^36^. Farrow identified differences in parent and grandparent reports of monitoring, child control, emotion regulation and restriction for weight^36^. However, Farrow’s study included caregivers of children aged 2-8 years old whereas our study just focused on caregivers of 2-4-year olds.

Regarding caregiver feeding practices, parents and grandparents scored highly (above 3.5/5) on feeding practices that provide structure to children’s eating such as modelling healthy eating, monitoring children’s intake and providing a healthy eating environment by making healthy foods available and limiting the amount of unhealthy food available. Additionally, both caregiver types scored highly on feeding practices that promote autonomy in children such as teaching them about nutrition and promoting balance and variety through encouraging preschoolers to try new and varied foods. This confirms what has been found in a study of Australian grandparents of 3-14-year-old grandchildren which reported grandparents using positive feeding practices, (which lead to favourable dietary behaviour) more frequently than negative, coercive ones ^37^. Other studies have found that grandparents do use coercive feeding practices with preschoolers such as using food to regulate emotions, pressuring children to eat and restricting foods for weight reasons ^9^. Feeding practices of grandparents may vary based on socioeconomic status and education level ^37^ and further work is needed to confirm whether demographic differences occur within a UK sample of grandparents.

### 5.3 Feeding Style

No significant associations were found between feeding styles and caregiver type. A range of feeding styles were demonstrated across both parents and grandparents. An authoritative approach, characterised by having high demands on children’s eating but being responsive to their preferences and needs, is associated with adequate dietary quality in preschool children ^17^ and is therefore considered the optimal approach. However less than a quarter of parents and grandparents in our study adopted this approach, suggesting a need to encourage both caregiver types to adopt this style. A previous review of grandparental feeding styles found that an indulgent feeding style was common in grandparents who care for their preschool aged grandchildren ^9^, however no study in this review used a validated tool to measure feeding styles. In contrast, using a validated tool to measure feeding styles, we found that over half of the grandparents in the current study did not adopt an indulgent feeding style. This highlights the importance of using tools that have been tested for their validity, reliability and sensitivity.

### 5.4 Associations with time grandparents spend caring for preschool aged grandchildren

Contrary to previous findings ^36,37^, we found few associations between the amount of time grandparents cared for their preschool aged grandchildren per week and dietary provision, feeding styles or practices. Only for vegetable provision in snacks was there a positive association, whereby grandparents who cared for their grandchildren for more hours provided greater quantities of vegetables as a snack to their preschool aged grandchildren. All grandparents in the current study were regular care providers to their grandchildren, providing a minimum of one day per week of care. Grandparents who routinely care for their grandchildren have reported feeling more responsible for their grandchildren compared with if they only saw their grandchildren occasionally ^35^. Further work is needed to explore the difference between feeding behaviours of those grandparents who routinely provide care compared with those that do so on a more casual basis.

Overall, the results of this study highlight the need to ensure that strategies to promote healthy eating in young children also target and reach grandparents who regularly provide childcare to their preschool aged grandchildren. Strategies are needed to encourage grandparents to reduce the amount of sodium and saturated fat in their meals whilst increasing the amounts of fruit and vegetables served at both mealtimes and as part of a snack. Grandparents should be supported to continue using those feeding practices that promote healthy eating in young children whilst being encouraged to use a more authoritative feeding style. Nevertheless, the similarities between the current, unrelated parent and grandparent feeding behaviours suggest that the content of interventions and policies designed with parents as the main audience, may not need to be adapted specifically for grandparents. Instead, there is a need to consider how strategies and public health messages can be altered to engage grandparents and explore what format of intervention grandparents would be most receptive to.

This study has several strengths. This is the first study to use a dietary assessment tool to assess UK grandparent’s food provision when caring for their preschool-aged grandchildren and is the first to use a validated tool to capture UK grandparental feeding styles. The results therefore provide an insight into the ways in which grandparents may influence young children’s eating behaviour. This study also benefits from looking at independent and unrelated parents and grandparents. Previously, correlations have been found between related mothers and grandmothers feeding practices ^45^ suggesting an intergenerational nature of feeding behaviours. By focusing on unrelated caregivers this potential confounding effect has been removed, strengthening the validity of the findings that grandparents who regularly act as caregivers to their preschool aged grandchildren use similar approaches to parents. Several limitations are also of note. As mentioned in the data analysis section, a power calculation was not carried out for this exploratory study. Due to the modest sample size the current results should be interpreted as exploratory and with caution. We adopted an appropriate, more conservative approach with the analysis that does not require certain assumptions to be met. Given the lack of previous studies in this area of research, the current study provides important details for future investigations, including sample size calculations. Moreover, the subscale “teaching about nutrition” (CFPQ) demonstrated poor internal consistency for parents and so these data should be interpreted with caution. Recruitment, particularly of grandparents was challenging, and the recruitment rates, in general, re-iterate the difficulty of recruiting participants to complete food records. Although a number of different methodological decisions were made to promote recruitment and completion of the food record e.g. using retrospective vs prospective methods, using an estimated vs weighed approach and using an intuitive online tool, further strategies to reach and engage grandparents and to increase participant compliance with food records are warranted. The recruitment strategy did result in a wide demographic participant pool with caregivers of different household incomes, weight status, and age but the sample was predominantly White British meaning that results are not generalisable to other ethnicities or outside of the UK. The use of food records also has its limitations. First, they rely on participant’s being able to accurately recall the food and drinks they provided and second, they could be subject to demand characteristics, whereby participants either selectively recall items or provide different food and drink items on diary days to appear healthier. myfood24^®^ has been specifically designed to promote accurate recall and, the dietary provision results of both caregivers might suggest that participants were minimally affected by demand characteristics.

### 5.5 Conclusions

Overall, very few differences were found between parents’ and grandparents’ dietary provision, feeding practices and feeding styles when caring for preschool-aged children. The results of this study have two key implications; first strategies to promote healthy eating in young children should be expanded to also target grandparents who act as informal caregivers to preschool-aged children. Second, those strategies that have been designed or reach mostly parents do not need to be adapted in content for grandparents. Instead, it is necessary to explore how receptive grandparents would be to receiving support and how best to promote their engagement.

## Figures and Tables

**Table 1:** Sample characteristics

		Grandparents (n=44)		Parents (n=72)	
		Mean	SD	Mean	SD
Caregiver Age*		61	6	31	5
Caregiver BMI**		26.8	5.1	26	0 6.6
**Hours per week Grandparents care for 2-4-year-old**		15.7	8.7		
		**N**	**%**	**N**	**%**
**Caregiver**	Male	6	14	0	0
**Gender**	Female	38	86	72	100
**Caregiver**	£0-£10,000	5	11.4	3	4.2
**Household**	£10,000-£20,000	8	18.2	7	9.7
**Income**	£20,000-£30,000	6	13.6	19	26.4
	£30,000-£40,000	10	22.7	21	29.2
	£40,000+	12	27.3	20	26.4
	Missing	3	6.8	3	4.2
**Caregiver Ethnicity**	White	40	91	61	85
	Asian or Asian British	3	7	8	4
	Black or Black British	1	2	0	0
	Mixed Ethnic Group	0	0	3	11
**Child Age**	2	15	34.1	18	25.0
	3	16	36.4	35	48.6
	4	13	29.5	19	26.4
**Child Gender**	Male	18	40.9	31	43.1
	Female	26	59.1	41	56.9
**Child BMI**	Healthy weight	16	36.4	21	29.2
	Overweight	8	18.2	8	11.1
	Obese	6	13.6	12	16.7
	Underweight	0	0	7	8.3
	Missing	14	31.8	25	34.7

* Missing data for 2 grandparents and 1 parent

** Missing data for 7 grandparents and 10 parents

**Table 2:** The nutritional content of meals (breakfast, lunch and evening meal) provided to children by parents and grandparents

	Breakfast			Lunch			Evening Meal		
	Parents (n=72)	Grandparents (n=37)	Mann-Whitney U Test	Parents (n=71)	Grandparents (n=40)	Mann-Whitney U Test	Parents (n=72)	Grandparents (n=34)	Mann-Whitney U Test
	Mean (SD)	Mean (SD)		Mean (SD)	Mean (SD)		Mean (SD)	Mean (SD)	
**Energy (kcal)**	197 (113)	232 (99)	U=1633, p=0.054	393 (240)	351 (198)	U=1240, p=0.269	323 (201)	341 (149)	U=1136, p=0.551
**Total Sugar (g)**	17 (27)	15 (11)	U=1463, p=0.404	15 (11)	18 (20)	U=1450, p=0.854	12 (10)	14 (11)	U=1380, p=0.291
**Sat Fat (g)**	5 (22)	1 (4)	U=1195, p=0.192	10 (16)	12 (16)	U=1513, p=0.547	15 (28)	19 (27)	U=1380 p=0.248
**Sodium (mg)**	267 (322)	246 (231)	U=1358, p=0.870	621(386)	545 (419)	U=1186, P=0.151	539 (383)	435 (320)	U=1026, p=0.179

SD = Standard deviation

**Table 3:** The amount of fruit and vegetables served at mealtimes (breakfast, lunch and evening meal) to children by parents and grandparents

	Breakfast			Lunch			Evening Meal		
	Parents (n=72)	Grandparents (n=37)	Mann-Whitney U Test	Parents (n=71)	Grandparents (n=40)	Mann-Whitney U Test	Parents (n=72)	Grandparents (n=34)	Mann-Whitney U Test
	Mean (SD)	Mean (SD)		Mean (SD)	Mean (SD)		Mean (SD)	Mean (SD)	
**Total Fruit (g)**	23 (37)	20 (45)	U=1228, p=0.406	27 (47)	29 (52)	U=1468, p=0.930	20 (45)	19(43)	U=1038, p=0.870
**Total Veg (g)**	0.3 (3)	0 (0)	U=1314, p=0.473	13(38)	25 (35)	U=1868, p=0.002*	49 (68)	44 (56)	U=1046, p=0.945

SD = Standard deviation

**Table 4:** The average nutritional content of snacks provided by parents and grandparents over the 24 hour period

	Snacks		
	Parents	Grandparents	Two sample T test
	Mean (SD)	Mean (SD)	
**Energy (Kcal)**	110 (63)	120 (69)	U=1498, p=0.416
**Total Sugar (g)**	11 (8)	11 (7)	U=1391, p=0.888
**Sat Fat (g)**	2 (9)	3 (8)	U=1439, p=0.441
**Sodium (mg)**	93 (101)	106 (136)	U=1428, p=0.706

SD = Standard deviation

**Table 5:** Provision of fruit, vegetables and discretionary food provided by parents and grandparents as snacks

	Parent (N=70)	Grandparent (N=38)	Chi Squared Test of Significance
	n (%)	n (%)	
**Snacks included fruit**			
**Yes**	46 (66%)	23 (61%)	X^2^ =(1, 108) = 0.287, p=0.592
**No**	24 (34%)	15 (39%)	
**Snacks included Vegetables**			
**Yes**	3 (4%)	4 (11%)	X^2^ =(1, 108) = 1.582, p=0.208
**No**	67 (96%)	34 (89%)	
**Snacks included discretionary food items**			
**Yes**	45 (64%)	23 (61%)	X^2^ =(1, 108) = 0.149, p=0.699
**No**	25 (36%)	15 (39%)	

**Table 6:** The amount of fruit, vegetables and discretionary food provided by parents and grandparents for snacks

	Parents	Grandparents	Mann-Whitney U Test
	Mean (SD)	Mean (SD)	
**Discretionary food items per snack occasion (grams)**	14 (17)	14 (19)	U=1309, p=0.889
**Fruit per snack occasion (grams)**	38 (47)	33 (40)	U=1262, p=0.652
**Vegetables per snack occasion (grams)**	1.0 (5)	4.0 (13)	U=1383, p=0.477

SD = Standard deviation

**Table 7:** Parent (n= 72) and grandparent (n = 44) feeding styles

	Authoritative (%)	Authoritarian (%)	Indulgent (%)	Uninvolved (%)
**Parent**	10 (14%)	30 (42%)	20 (28%)	12 (16%)
**Grandparent**	10 (23%)	8 (18%)	18 (41%)	8 (18%)

**Table 8:** Parent and Grandparent feeding practices

	Grandparents (n=44)	Parents (n=72)	Mann Whitney Test
	Mean (SD)	Mean (SD)	
Child Control	2.6 (0.7)	2.7(0.8)	U=1466.5, p=0.502
Emotion Regulation	2.0 (0.6)	2.3 (0.9)	U = 1433, p=0.385
**Food as Reward**	**2.2 (1.1)**	**2.8 (1.2)**	U= 1155.5, p=<0.05*
**Balance & Variety**	**4.5 (0.5)**	**4.7 (0.5)**	U=1178.5, p=<0.05*
Involvement	3.6 (1.1)	3.7(1.1)	U= 1490.5, p= 0.592
Modelling	4.3 (0.7)	4.4 (0.7)	U=1498.5, p=0.621
Monitoring	4.1 (1.0)	4.3 (0.8)	U = 1520, p=0.709
Pressure	2.9 (1.0)	3.2 (1.0)	U=1351, p=0.183
Restriction for Health	3.2 (1.0)	3.5 (1.0)	U=1283, p=0.086
Restriction for Weight	2.2 (0.7)	2.3 (0.8)	U=1633, p=0.780
Teaching about Nutrition	3.7(0.9)	3.8 (0.8)	U=1522, p=0.722
**Environment**	**4.1 (0.7)**	**3.6 (0.8)**	U= 2176, p=<0.05*

SD = Standard deviation

**Table 9:** Spearman’s rho correlations between grandparent’s meal and snack provision and time spent caring for grandchild each week

	No. of hours
**Breakfast**	
Kcal	-0.177
Total Sugar	-0.184
Saturated Fat	0.131
Sodium	-0.144
Grams of fruit	-0.109
Grams of veg	Not served by grandparents
**Lunch**	
Kcal	0.029
Total Sugar	-0.046
Saturated Fat	-0.143
Sodium	0.053
Grams of fruit	-0.122
Grams of veg	-0.172
**Evening Meal**	
Kcal	-0.255
Total Sugar	0.096
Saturated Fat	-0.275
Sodium	-0.282
Grams of fruit	0.179
Grams of veg	-0.111
**Snacks**	
Grams of cakes biscuits, chocolate and savoury items in snacks	-0.172
Grams of fruit in snacks	-0.005
Grams of vegetables in snacks	0.358*
Kcal in snacks	0.170
Sugar in snacks	0.043
Saturated fat in snacks	0.279
Salt in snacks	0.152

* Significant p<0.05

**Table 10:** Grandparents feeding style and the number of hours spent caring for their grandchild per week

	Mean hours per week	Standard Deviation
**Authoritative**	18.7	4.3
**Authoritarian**	19.1	14.3
**Indulgent**	13.6	7.5
**Uninvolved**	12.0	7.1

## References

[1] Department for Education Childcare and Early Years Survey of Parents in England.

[2] Wheelock J, Jones K (2021). ‘Grandparents Are the Next Best Thing’: Informal Childcare for Working Parents in Urban Britain.

[3] Jappens M, Van Bavel J (2012). Regional family norms and child care by grandparents in Europe. Demogr Res.

[4] Geurts T, Van Tilburg T, Poortman AR, Dykstra PA (2015). Child care by grandparents: Changes between 1992 and 2006. Ageing Soc.

[5] Buchanan A, Rotkirch A (2018). Twenty-first century grandparents: global perspectives on changing roles and consequences. Contemp Soc Sci.

[6] Department for Education (2018). Childcare and Early Years Survey of Parents in England, 2018.

[7] Hank K, Buber I (2008). Grandparents Caring for their Grandchildren: Findings From the 2004 Survey of Health, Ageing, and Retirement in Europe.

[8] Savage JS, Fisher JO, Birch LL (2007). Parental influence on eating behavior: conception to adolescence. J Law Med Ethics.

[9] Marr C, Reale S, Breeze P, Caton SJ (2020). Grandparental dietary provision, feeding practices and feeding styles when caring for preschool-aged grandchildren: A systematic mixed methods review. Obes Rev.

[10] Brunstrom JM, Mitchell GL, Baguley TS (2005). Potential early-life predictors of dietary behaviour in adulthood: a retrospective study. Int J Obes.

[11] Skinner JD, Carruth BR, Bounds W, Ziegler P, Reidy K (2002). Do food-related experiences in the first 2 years of life predict dietary variety in school-aged children?. J Nutr Educ Behav.

[12] Rollins BY, Loken E, Birch LL (2010). Stability and change in snack food likes and dislikes from 5 to 11 years. Appetite.

[13] Nicklaus S, Boggio V, Chabanet C, Issanchou S (2004). A prospective study of food preferences in childhood.

[14] Hughes SO, Power TG, Orlet Fisher J, Mueller S, Nicklas TA (2005). Revisiting a neglected construct: parenting styles in a child-feeding context. Appetite.

[15] Baranowski T, O’Connor T, Hughes S (2013). Houston… We have a problem! measurement of parenting. Child Obes.

[16] Vollmer RL, Mobley AR (2013). Parenting styles, feeding styles, and their influence on child obesogenic behaviors and body weight. A review. Appetite.

[17] Arlinghaus KR, Vollrath K, Hernandez DC (2018). Authoritative parent feeding style is associated with better child dietary quality at dinner among low-income minority families. Am J Clin Nutr.

[18] Vollmer RL (2019). Parental feeding style changes the relationships between children’s food preferences and food parenting practices: The case for comprehensive food parenting interventions by pediatric healthcare professionals. J Spec Pediatr Nurs.

[19] Blissett J (2011). Relationships between parenting style, feeding style and feeding practices and fruit and vegetable consumption in early childhood. Appetite.

[20] Galloway AT, Fiorito L, Lee Y, Birch LL (2005). Parental pressure, dietary patterns, and weight status among girls who are “picky eaters. J Am Diet Assoc.

[21] Fletcher A, Wolfenden L, Wyse R, Bowman J, McElduff P, Duncan S (2013). A randomised controlled trial and mediation analysis of the “Healthy Habits”, telephone-based dietary intervention for preschool children. Int J Behav Nutr Phys Act.

[22] Ostbye T, Krause KM, Stroo M (2012). Parent-focused change to prevent obesity in preschoolers: results from the KAN-DO study. Prev Med (Baltim).

[23] Haycraft E, Witcomb GL, Farrow C (2020). The Child Feeding Guide: A digital health intervention for reducing controlling child feeding practices and maternal anxiety over time. Nutr Bull.

[24] Reale S, Kearney CM, Hetherington MM (2018). The Feasibility and Acceptability of Two Methods of Snack Portion Control in United Kingdom (UK) Preschool Children: Reduction and Replacement. Nutrients.

[25] NHS Change4Life Food Facts Five a Day.

[26] Public Health England Sugar facts leaflet (Sugar Swaps).

[27] Infant and Toddler Forum Portion Sizes for Toddlers.

[28] NHS Change4Life Food Facts 100 Calorie Snacks.

[29] Eli K, Howell K, Fisher PA, Nowicka P (2016). A question of balance: Explaining differences between parental and grandparental perspectives on preschoolers’ feeding and physical activity. Soc Sci Med.

[30] Mena NZ, Gorman K, Dickin K, Greene G, Tovar A (2015). Contextual and Cultural Influences on Parental Feeding Practices and Involvement in Child Care Centers among Hispanic Parents. Child Obes.

[31] Lindsay AC, Sussner KM, Greaney ML, Peterson KE (2009). Influence of Social Context on Eating, Physical Activity, and Sedentary Behaviors of Latina Mothers and Their Preschool-Age Children. Heal Educ Behav.

[32] Dwyer J, Needham L, Simpson JR, Heeney ES (2008). Parents report intrapersonal, interpersonal, and environmental barriers to supporting healthy eating and physical activity among their preschoolers. Appl Physiol Nutr Metab.

[33] Toftemo I, Glavin K, Lagerløv P (2013). Parents’ views and experiences when their preschool child is identified as overweight: a qualitative study in primary care. Fam Pract.

[34] Jiang J, Rosenqvist U, Wang H, Greiner T, Lian G, Sarkadi A (2007). Influence of grandparents on eating behaviors of young children in Chinese three-generation families. Appetite.

[35] Pankhurst M, Mehta K, Matwiejczyk L (2019). Treats are a tool of the trade: An exploration of food treats among grandparents who provide informal childcare. Public Health Nutr.

[36] Farrow C (2014). A comparison between the feeding practices of parents and grandparents. Eat Behav.

[37] I Jongenelis M, Morley B, Pratt IS, Talati Z (2020). Diet quality in children: A function of grandparents’ feeding practices?. Food Qual Prefer.

[38] Andrade C (2018). Internal, external, and ecological validity in research design, conduct, and evaluation. Indian J Psychol Med.

[39] Christensen T, Riis AH, Hatch EE (2017). Costs and efficiency of online and offline recruitment methods: A web-based cohort study. J Med Internet Res.

[40] Klesges RC, Klesges LM, Brown G, Frank GC (1987). Validation of the 24-hour dietary recall in preschool children. J Am Diet Assoc.

[41] Carter M, Albar S, Morris M (2015). Development of a UK Online 24-h Dietary Assessment Tool: myfood24. Nutrients.

[42] Cade JE (2017). Measuring diet in the 21st century: use of new technologies. Proc Nutr Soc.

[43] Musher-Eizenman D, Holub S (2007). Comprehensive Feeding Practices Questionnaire: Validation of a New Measure of Parental Feeding Practices. J Pediatr Psychol.

[44] Russell CG, Haszard JJ, Taylor RW, Heath A-LM, Taylor B, Campbell KJ (2018). Parental feeding practices associated with children’s eating and weight: What are parents of toddlers and preschool children doing?. Appetite.

[45] Metbulut AP, Ozmert EN, Teksam O, Yurdakok K (2018). A comparison between the feeding practices of parents and grandparents. Eur J Pediatr.

[46] Shloim N, Edelson LR, Martin N, Hetherington MM (2015). Parenting Styles, Feeding Styles, Feeding Practices, and Weight Status in 4-12 Year-Old Children: A Systematic Review of the Literature. Front Psychol.

[47] Hughes SO, Patrick H, Power TG, Fisher JO, Anderson CB, Nicklas TA (2007). The Impact of Child Care Providers??? Feeding on Children???s Food Consumption. J Dev Behav Pediatr.

[48] Hughes SO, Shewchuk RM, Baskin ML, Nicklas TA, Qu H (2008). Indulgent Feeding Style and Children’s Weight Status in Preschool. J Dev Behav Pediatr.

[49] O’Connor TM, Hughes SO, Watson KB (2010). Parenting practices are associated with fruit and vegetable consumption in pre-school children. Public Health Nutr.

[50] World Health Organization (2018). Guidelines: Saturated Fatty Acid and Trans-Fatty Acid Intake for Adults and Children (Draft Issued for Public Consultation InMay 2018).

[51] Salt: the facts - NHS https://www.nhs.uk/live-well/eat-well/salt-nutrition/.

[52] The Caroline Walker Trust Eating well for 1-4 year olds.

[53] Public Health England (2016). The Eatwell Guide.

[54] Speirs KE, Braun B, Zoumenou V, Anderson EA, Finkbeiner N (2009). Grandmothers’ Involvement in Preschool-Aged Children’s Consumption of Fruits and Vegetables. ICAN Infant, Child, Adolesc Nutr.

[55] Yue A, Zhang N, Liu X (2018). Do Infant Feeding Practices Differ Between Grandmothers and Mothers in Rural China? Evidence From Rural Shaanxi Province. Fam Community Health.

[56] Change4Life 100 Calorie Snacks.

[57] Reale S, Simpson RM, Marr C (2019). Snack portion sizes for preschool children are predicted by caregiver portion size, caregiver feeding practices and children’s eating traits. Nutrients.

